# Transient symptomatic hyperglycaemia secondary to inhaled fluticasone propionate in a young child

**DOI:** 10.1186/s12890-016-0170-z

**Published:** 2016-01-13

**Authors:** Mara Lelii, Nicola Principi, Susanna Esposito

**Affiliations:** Department of Pathophysiology and Transplantation, Pediatric Highly Intensive Care Unit, Università degli Studi di Milano, Fondazione IRCCS Ca’ Granda Ospedale Maggiore Policlinico, Via Commenda 9, 20122 Milan, Italy

**Keywords:** Asthma, Fluticasone propionate, Hyperglycaemia, Wheezing

## Abstract

**Background:**

Inhaled corticosteroids (ICSs) are currently used to prevent and treat asthma and recurrent wheezing attacks in children. Fluticasone propionate (FP) is one of the most commonly prescribed ICSs because it is considered effective and well tolerated.

**Case presentation:**

A male infant of approximately 1 year of age, who was born to parents without relevant clinical problems or family histories including diabetes, was brought to our attention for recurrent wheezing. When he was approximately 2 years old, a regular daily inhaled treatment with FP given using a spacer was prescribed. With this therapy, the child obtained good control of his symptoms with no further recurrences, but after approximately 2 months of treatment he was admitted to the emergency room because he was whining and agitated and exhibited increased diuresis and water intake. Laboratory tests revealed hyperglycaemia (181 mg/dL), mild glycosuria, blood alkalosis (pH 7.49), a bicarbonate level of 31 mmol/L, a pCO_2_ level of 39 mmHg, a serum sodium level of 135 mEq/L and a serum potassium level of 3.5 mEq/L. The parents confirmed that the recommended dose of FP had been administered with no increase in the amount of drug. The child was immediately treated with endovenous infusion of physiological saline for 24 h, and his glycaemic levels as well as venous blood gas analysis returned to normal, with an absence of glucose in the urine. Oral glucose tolerance test results and glycated haemoglobin levels were normal. Monitoring of blood glucose levels before and after meals for three consecutive days did not reveal any further increase above normal levels. He was discharged with a diagnosis of transient symptomatic hyperglycaemia during ICS therapy and the suggestion to replace his inhaled FP therapy with oral montelukast. Montelukast was continued for 6 months; during this time, the child did not present any other hyperglycaemia episodes.

**Conclusions:**

Although there is no evidence of causation, this case report represents an interesting and unusual description of paediatric transient symptomatic hyperglycaemia after treatment with inhaled FP and highlights the importance of considering this potential adverse event and the necessity of informing parents of the possible clinically relevant risks associated with this drug.

## Background

As in adults, inhaled corticosteroids (ICSs) are currently used in children to prevent and treat asthma and recurrent wheezing attacks. Fluticasone propionate (FP), either alone or in association with other bronchodilators, is one of the most commonly prescribed ICSs because it is considered effective and well tolerated [1]. Due to its low systemic bioavailability, FP has been associated with a lower rate of adverse reactions in comparison to other ICSs including budesonide [2]. Moreover, at the recommended doses of 100–200 μg/day FP administration can significantly reduce the incidence of wheezing exacerbation and improve respiratory symptoms during acute attack without any relevant adverse effects even in young children [1]. Only long-term treatment with significantly higher than recommended dosages has been associated with clinical complications, including growth retardation, bone osteoporosis, and acute adrenal crisis with hypoglycaemia [3, 4]. Moreover, although a number of studies have reported that adults receiving ICS, including FP, can develop diabetes or experience the progression of already diagnosed diabetes [5], no case of symptomatic hyperglycaemia has been reported in children (Table 1). Recently, we diagnosed the first known case of transient symptomatic hyperglycaemia in a child with recurrent wheezing and no predisposing factor for poor glucose tolerance who was treated with FP. We report this case to highlight the importance of considering this potential adverse event during FP treatment in children.

**Table 1:** Main characteristics of a child who developed transient symptomatic hyperglycaemia during treatment with a moderate dose of inhaled fluticasone propionate (FP)

Characteristic	Patient
Age	2 years
Gender	Male
Previous personal history	Recurrent asthmatic bronchitis and atopic dermatitis
Familial history	Father with allergic asthma induced by inhaled allergens, uncle with coeliac disease, no other history of immune-mediated disease in the family
Allergic tests	IgE levels of 124 KU/L, skin prick testing negative for standard food and inhaled allergens
Therapies in the past year	Repeated courses of 100 μg inhaled FP twice a day for 7 days and then 50 μg twice a week for 3 days during acute events; oral corticosteroids in three cases
Therapy at hyperglycaemia onset	100 μg inhaled FP twice a day from October 1, 2014, to November 27, 2014
Symptoms at emergency room admission	Whining and agitation with increased diuresis and water intake
Clinical signs at admission	Whining and agitation, no sign suggestive of acute infection, normal respiratory evaluation
Laboratory evaluation	
Glycaemia	181 mg/dL
Glycosuria	Present
Blood gas analysis	pH 7.49, bicarbonates 31 mmol/L, pCO2 39 mmHg, sodium 135 mEq/L, potassium of 3.5 mEq/L
Oral glucose tolerance test	Normal
Glycated haemoglobin	7 %
Treatment	Endovenous infusion of physiological saline for 48 h
Persistence of symptoms	6 h
Outcome	Replacement of inhaled FP therapy with oral 4 mg montelukast once a day, no further hyperglycaemia episodes after 6 months

## Case presentation

A male infant of approximately 1 year of age, who was born to parents without relevant clinical problems or family histories including diabetes, was brought to the Outpatient Clinic for Pediatric Infectious Diseases of the University of Milan, Fondazione IRCCS Ca’ Granda Ospedale Maggiore Policlinico, Milan, Italy, for recurrent wheezing. Staring from the age of 6 months, he had suffered from monthly episodes of upper respiratory infection associated with severe wheezing at every occasion. At admission, his general condition was good, and there were no signs or symptoms of infection or bronchial obstruction.

Laboratory tests revealed increased IgE levels (124 KU/L), and skin prick testing for standard food and inhaled allergens was negative. Serum immunoglobulins (Ig and IgG subclasses) were within normal ranges. On the basis of the patient’s clinical history, in case of an acute event, the administration of 100 μg of inhaled FP administered using a spacer twice a day for 7 days and then 50 μg twice a week for 3 days was suggested, with inhaled salbutamol as needed. However, despite this protocol, over the following 12 months the child experienced 6 additional episodes of wheezing, 3 of which were severe enough to require oral corticosteroid administration to be controlled. Consequently, at the beginning of the following fall season (i.e., 6 months after the last administration of oral corticosteroids), when he was approximately 2 years old, a regular daily inhaled treatment with FP given using a spacer was prescribed. He received 100 μg of inhaled FP twice a day starting on October 1, 2014, and the indication was to continue this treatment up until February 28, 2015.

With this therapy, the child obtained good control of his symptoms with no further recurrences, but after approximately 2 months of treatment he was admitted to the emergency room because he was whining and agitated and exhibited increased diuresis and water intake. Laboratory tests revealed hyperglycaemia (181 mg/dL), mild glycosuria, blood alkalosis (pH 7.49), a bicarbonate level of 31 mmol/L, a pCO_2_ level of 39 mmHg, a serum sodium level of 135 mEq/L and a serum potassium level of 3.5 mEq/L. The parents confirmed that the recommended dose of FP had been administered with no increase in the amount of drug. The child was immediately treated with endovenous infusion of physiological saline for 24 h, and his glycaemic levels as well as venous blood gas analysis returned to normal, with an absence of glucose in the urine. Oral glucose tolerance test (OGTT) results were normal and glycated haemoglobin (HbA1c) level was 7 %. Monitoring of blood glucose levels before and after meals for three consecutive days did not reveal any further increase above normal levels. He was discharged with a diagnosis of transient symptomatic hyperglycaemia during ICS therapy and the suggestion to replace his inhaled FP therapy with 4 mg oral montelukast once a day. Montelukast was continued for 6 months; during this time, the child did not present any other hyperglycaemia episodes.

## Conclusions

Because of their topical application, ICSs have a substantially better therapeutic index than oral corticosteroids and have almost entirely replaced these drugs in the prevention and treatment of asthma and wheezing in both children and adults [1, 5]. FP pharmacokinetic characteristics suggest a very low risk of systemic adverse events [1]. In normal subjects, FP bioavailability is limited to approximately 1 %, the lowest value among all of the commonly used ICSs, and its high first-pass metabolism ensures a prompt inactivation of the amount of drug that is swallowed during administration. Practically, the risk of systemic adverse events with doses useful to control bronchial obstruction is marginal and is probably even lower in patients with moderate or severe asthma, in whom factors such as airflow obstruction and ventilation-perfusion mismatch could alter drug deposition in the lung and change systemic absorption. Previous studies on ICSs efficacy have also included evaluation of their safety with routine blood chemistry assessment and haematological testing (that include blood glucose levels), showing that doses used for wheezing prevention (including FP at 200 μg/day) have no clinically relevant side effects in pediatric age [6–8]. In children, problems regarding glucose metabolism have been reported only when significantly higher than recommended FP doses were used. Todd et al. have described three children ranging in age from 7 to 9 years old who received inhaled FP at a dosage of 500–2000 μg/day for between 5 months and 5 years and who were admitted to the hospital for seizures secondary to severe hypoglycaemia (blood glucose between 23.4 and 32.4 mg/dL) [3]. A similar finding has been reported by Drake et al. in four children treated with FP at a dose of 500–1500 μg/day for between 15 months and 5 years [4]. In all cases, hypoglycaemia was a consequence of iatrogenic adrenal suppression [3, 4].

Increased blood glucose concentration is a common complication of oral corticosteroid administration because these drugs increase gluconeogenesis and decrease glucose uptake in the liver and adipocytes by decreasing insulin binding [9]. In adults, ICS administration has been associated with a moderate risk of increased blood glucose concentrations only when administered at higher than recommended doses or when administered to patients already diagnosed as diabetic [9]. The child in the current report had no predisposing factor for poor glucose tolerance, as evidenced by normal values of OGTT and HbA1c, which were assessed within a few days after hospital admission. The development of symptomatic hyperglycaemia is surprising and difficult to explain. The patient did not receive oral corticosteroids close to the administration of FP. Due to the presence of glycosuria, blood alkalosis and clinical symptoms, the hypothesis is that in the month prior to admission his blood glucose levels were even higher than those detected by us at admission in the Emergency Room. However, we did not evaluate blood glucose and HbA1c concentrations prior to the initiation of FP and we did not know whether these values were in the higher normal range before the beginning of FP treatment. Moreover, FP was prescribed at an adequate dose and it was administered for a relatively short period of time. The parents confirmed that FP was administered according to recommendations, but the reported level of compliance of parents has a very low accuracy. However, although there is no evidence of causation, this interesting and unusual case report highlights the importance of informing parents of the possible clinically relevant risks associated with the use of this drug.

### Consent

This case report has been approved by the Ethics Committee of Fondazione IRCCS Ca’ Granda Ospedale Maggiore Policlinico, Milan, Italy. Written informed consent was obtained from the patient’s parents for publication of this case report. A copy of the written consent is available for review from the Editor-in-Chief of this journal.

## Abbreviations

ICS: Inhaled corticosteroids
FP: Fluticasone propionate
OGTT: Oral glucose tolerance test
HbA1c: Glycated haemoglobin
